# Optimizing the Construction and Activation of 3D-Printed Electrochemical Sensors: An Experimental Design Approach for Simultaneous Electroanalysis of Paracetamol and Caffeine

**DOI:** 10.1021/acsomega.4c08593

**Published:** 2025-01-06

**Authors:** José G. A. Rodrigues, Tárcila M. N. Silva, Sidnei B. Gomes Junior, Antonio A. L. Marins, Gabriel F. S. dos Santos, Rafael Q. Ferreira, Jair C. C. Freitas

**Affiliations:** † Laboratory of Carbon and Ceramic Materials (LMC), Department of Physics, Center of Exact Sciences, Federal University of Espírito Santo, Vitória 29075-910, Espírito Santo, Brazil; ‡ Electrochemistry Research and Development Laboratory, Department of Chemistry, Center of Exact Sciences, Federal University of Espírito Santo, Vitória 29075-910, Espírito Santo, Brazil; § Multiuser Instrumentation Laboratory, Center of Exact Sciences, Federal University of Espírito Santo, Vitória 29075-910, Espírito Santo, Brazil; ∥ Center of Research, Innovation and Development of Espirito Santo, Ladeira Eliezer Batista, Cariacica 29140-130, Espírito Santo, Brazil

## Abstract

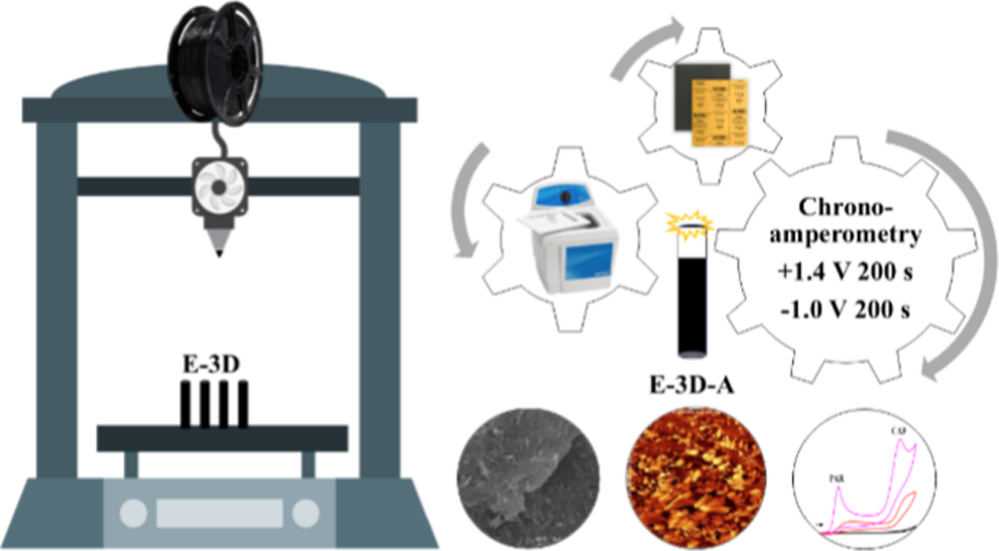

This work presents an optimization of the construction, treatment, and activation of 3D-printed electrochemical sensors (E-3D). For this, was used a 2^3^-full factorial design examining three key variables at two levels: electrode height, electrode diameter, and printing speed. Moreover, it evaluates various physical, chemical, and electrochemical methods to treat and activate the E-3D surface. The techniques of electrochemical impedance spectroscopy and cyclic voltammetry (CV) shows that the sequential physical, chemical, and electrochemical treatments lead to the highest treatment efficiency and activation. Raman spectroscopy and atomic force microscopy characterize untreated and treated E-3D sensor surfaces. The optimal treatment and activation methodology was applied to the electroanalysis of paracetamol (PAR) and caffeine (CAF) simultaneously using CV and differential pulse anodic stripping voltammetry (DPASV). DPASV measurements reveal limits of detection of 0.44 and 0.58 μmol L^–1^ in a 0.5 mol L^–1^ H_2_SO_4_ medium for PAR and CAF, respectively, with the treated and activated E-3D sensor. The principal achievement of this work was emphasizing the critical role of surface treatment and activation in enhancing the performance of the developed electrodes, thereby advancing technological applications of 3D-printed electrochemical sensors.

## 1 Introduction

Three-dimensional (3D) printing technology has proven to be a valuable tool in a various research and applications. It has been combined with electrochemical techniques to construct electrochemical cells and produce electrodes for batteries, supercapacitors, and electrochemical sensors (refs (1–4)). In this sense, 3D printing has emerged as a promising technology for advancing of electrochemical devices owing to its large-scale production capacity, low energy consumption, and ability to obtain different shapes and sizes while maintaining accessibility. Additionally, this technology allows for miniaturization and free design (refs (5 and 6)). Several 3D printing technologies are currently available, highlighting the extrusion. Between the extrusion methods, fused deposition modeling (FDM) is the most prevalent employed. In this process, polymers with thermoplastic properties, such as polylactic acid (PLA) or acrylonitrile butadiene styrene (ABS) are heated to a semimolten state before being extruded through the distributor nozzle and deposited onto a surface (ref (7)). Once deposited, the material solidifies, forming a layer that is stacked on top of the previous layer. This process is repeated layer-by-layer until the object is completely printed. FDM is the simplest, cheapest, and most popular 3D printing technology (ref (8)). The use of 3D printing technology in the field of electrochemical sensors has emerged as an alternative to commercial electrodes. To fabricate the sensors, PLA or ABS filaments are typically used, which need to contain a conductive material. This is usually achieved by incorporating carbon-based materials such as carbon black, graphite, and graphene (ref (9)).

There are several reports in the literature of 3D-printed sensors used in various fields, including forensics (ref (10)), pesticide detection (refs (11 and 12)), multivariate analysis for determining coffee quality (refs (13 and 14)), and biological tyrosine detection (ref (15)). Although 3D-printed electrochemical sensors are highly applicable in various areas, they require pretreatments or modifications of the electrode surface to reduce the amount of insulating polymer and improve the performance of the analytical electrochemical sensor (refs (10 and 13)).

In this sense, it is possible to employ 3D sensors associated with electroanalytical techniques, such as differential pulse voltammetry (DPV), to monitor emerging contaminants. Moreover, this printed sensor can achieve results compared to conventional and commercial sensors, reducing the cost of electroanalysis and making it more viable for monitoring and quantifying drugs (refs (11, 12, 16–20)). However, to improve the performance of the 3D sensor, it is important to treat their surface and enhance the analyte signal. Consequently, this study aims to optimize the construction, treatment, and activation of 3D-printed electrochemical sensors to analyze paracetamol (PAR) and caffeine (CAF).

## 2 Materials and Methods

### 2.1 Reagents and Solutions

Solutions were prepared using high-purity reagents and ultrapure water (resistivity of 18.2 MΩ cm at 25 °C) produced by a Sartorius Arium Mini-H_2_O MA-UV-T purification system (Germany). The reagents purchased were anhydrous caffeine (Sigma-Aldrich, USA), paracetamol (Sigma-Aldrich, USA), sulfuric acid (Fluka, Germany), and sodium hydroxide (Fisher Chemical, United Kingdom). Britton–Robinson (BR) buffer solutions with pH values of 1.0, 3.0, 5.0, 7.0, and 9.0 were employed, with pH adjustments made using a 2.0 mol L^–1^ NaOH solution.

### 2.2 Instruments and Apparatus

Voltammetric experiments were performed using a PGSTAT 128N AUTOLAB potentiostat/galvanostat (Metrohm, Switzerland) connected to a computer operating Nova 2.1.5 program (Metrohm). The electrochemical cell system (Figure S1, Supporting Information) comprised a 10 mL beaker equipped with a 3D-printed lid modified for a three-electrode configuration, featuring a lab-made Ag/AgCl (3.0 mol L^–1^ KCl) electrode and a platinum bar serving as reference and auxiliary electrodes, respectively. Moreover, 3D-printed electrodes (E-3Ds) were used as the working electrodes. These electrodes were fabricated using a Sethi3D S3 printer (from Brazil) and a commercial conductive filament composed of polylactic acid and carbon black (PLA-CB) from Protopasta (USA). The 3D designs were created using Tinkercad (https://www.tinkercad.com), and the electrodes were sliced and printed using Simplify3D. After printing the E-3Ds, thread-sealing tape was used on the electrode, leaving only the cross-sectional area exposed in the solution to establish the appropriate electrical connections.

### 2.3 Optimizing the Construction and Activation of E-3Ds

#### 2.3.1 Construction of E-3Ds

Design of experiments (DoE) was used to optimize the construction of the E-3Ds. Initially, the screening stage involved the use of a full factorial design 2^3^ to build the electrodes, studying the effects of three variables at two levels: electrode height (*h*), electrode diameter (*d*), and printing speed (*p*_s_). This resulted in 11 experiments (Table S1, Supporting Information). Only physical polishing of the electrode surface (with 600 and 1200 grift sandpaper) was used during the construction of the E-3Ds at this stage. The anodic peak current density in the presence of 1.0 mmol L^–1^ potassium ferricyanide in 0.5 mol L^–1^ H_2_SO_4_ was measured using the cyclic voltammetry (CV) technique. The working range was from 0.0 to +0.8 V, with a scan rate of 50 mV s^–1^. The data for the full factorial design 2^3^ was processed using the Statistica (version 14.0.1) software.

#### 2.3.2 Treatment and Activation of the E-3D

To enhance the electrochemical response, various methods of treating and activating the electrode surface were evaluated after optimizing the E-3D construction. Physical, chemical, and electrochemical treatments were studied as forms of treatment and activation. The samples were polished using 600 and 1200 grit sandpaper, immersed in a 0.5 mol L^–1^ NaOH solution for 30 min in an ultrasonic bath, and subjected to chronoamperometry with a potential of +1.4 V for 200 s followed by −1.0 V for 200 s in a 0.5 mol L^–1^ NaOH solution (ref (4)).

The voltammetric profile was analyzed using the CV technique in the presence of 1.0 mmol L^–1^ potassium ferricyanide in 0.5 mol L^–1^ H_2_SO_4_. The working range was from 0.0 to +0.8 V, with a scan rate of 50 mV s^–1^. Electrochemical impedance spectroscopy (EIS) was performed to further characterize the electrochemical response of the activated E-3Ds. The tests were conducted in the presence of 1 mmol L^–1^ potassium ferricyanide in 0.5 mol L^–1^ H_2_SO_4_ with a disturbance amplitude of 10 mV, over a frequency range from 100 kHz to 0.01 Hz with 5 points per decade.

### 2.4 Morphological and Structural Characterization of E-3Ds by SEM, Raman, and AFM

For comparison purposes, the morphological and structural characterization tests were carried out using the physically polished electrode (E-3D-P), the electrode without any form of treatment (E-3D-S), and the electrode treated under conditions optimized by DoE (E-3D-A). E-3D surfaces were characterized using scanning electron microscopy (SEM), using a JEOL JSM6610LV microscope. Surface characterization by atomic force microscopy (AFM) and Raman spectroscopy was performed using a WITec Raman-AFM microscope Alpha300 RA instrument (Oxford Instruments—WITec, Ulm, BW, Germany).

AFM images were acquired using amplitude-modulated AC mode (intermittent contact, phase imaging) under ambient conditions, with a 42 N/m spring constant and a resonance frequency of 285 kHz. Multiple sample regions were selected for topographic analysis, with each area scanned at a resolution of 640 pixels per line and a scan rate below 1 Hz, utilizing a 20× microscope objective (Zeiss EC Epiplan 20×/0.4). Raman spectra were obtained with a 532 nm laser, a 200 μm confocal aperture, and a G1: 600 grooves/mm grating (BLZ = 500 nm) for a spectral resolution of 1 cm^–1^. The measurements, performed under ambient conditions at 293 K, involved 0.5 s acquisition times and 50 accumulations. All data and imaging analyses were carried out using WITec’s Control FIVE 5.2 and Project FIVE 5.2 software.

### 2.5 Application of E-3Ds in Electroanalysis of PAR and CAF

#### 2.5.1 Electrochemical Behavior of PAR and CAF

After defining the optimized conditions for the construction, treatment, and activation of the E-3D, the electrochemical behavior of PAR and CAF was studied using the developed sensor at different pH values (from 1.0 to 9.0). For the pH study, a 0.5 mol L^–1^ H_2_SO_4_ solution and 0.1 mol L^–1^ BR buffer solution were used as the supporting electrolyte. The technique used was CV over a potential range from 0.3 to +1.8 V, with a scan rate of 50 mV s^–1^. Subsequently, the scan rate study was carried out by varying it from 10 to 200 mV s^–1^ using 0.5 mol L^–1^ H_2_SO_4_ as the supporting electrolyte.

#### 2.5.2 Optimization of DPASV Parameters

Differential pulse anodic stripping voltammetry (DPASV) analyses were conducted using a 0.5 mol L^–1^ H_2_SO_4_ electrolyte over a potential range from 0.5 to +1.65 V. The parameters related to the DPASV technique were optimized using multivariate analysis through DoE. The variables studied included step potential (*x*_1_), modulation amplitude (*x*_2_), preconcentration time (*x*_3_), preconcentration potential (*x*_4_), and stirring rate (*x*_5_). A 2^5–2^ fractional factorial design, as detailed in Table S2 (Supporting Information), was employed for this purpose. The variables were analyzed at two levels, resulting in 19 experiments. After the multivariate analysis, a modeling stage was conducted to develop a second-degree polynomial, enabling the application of the response surface methodology. Face central composite design (FCCD) was used during this stage. The data obtained in these experiments were processed using the Statistica (version 14.0.1) software.

#### 2.5.3 Calibration Curve Construction and Determination of Performance Characteristics

The linearity of the electrochemical response obtained with the developed electrodes was first evaluated over a wide concentration range: from 0.41 to 48.61 μmol L^–1^ for PAR and from 0.87 to 102.93 μmol L^–1^ for CAF, both in a 0.5 mol L^–1^ H_2_SO_4_ medium. After confirming the linearity of the electrochemical response within these ranges, lower concentration ranges were selected for PAR and CAF to construct the corresponding calibration curves. This was performed with a potential range from +0.50 to +1.65 V vs Ag/AgCl using optimized DPASV parameters. Subsequently, the main performance parameters were determined.

#### 2.5.4 Stability Test

The evaluation of the stability of the E-3D-A was conducted using DPASV with 50.0 μmol L^–1^ PAR and 100.0 μmol L^–1^ CAF in a 0.5 mol L^–1^ H_2_SO_4_ solution. The variation of the peak current (*I*_p_) was monitored as a function of the number of measurements (*n* = 25) in successive DPASV scans. The DPASV parameters included a preconcentration time of 150 s, a preconcentration potential of −0.60 V, a stirring rate of 1 rpm, a step potential of 25 mV, a modulation amplitude of 80 mV, a start potential of +1.20 V, and a stop potential of +1.60 V vs Ag/AgCl.

#### 2.5.5 Quantification of Paracetamol and Caffeine in Real Sample

Following the development of the methodology, its efficiency was assessed by quantifying both PAR and CAF in a pharmaceutical sample (PS) acquired from a local drugstore in Vitória-ES, Brazil. The sample was ground to a fine powder and macerated using a mortar and pestle. After this, the PS was dissolved in ultrapure water within a 500 mL volumetric flask. A recovery test was performed with the voltammetric method, employing the E-3D-A sensor to determine the PAR and CAF levels in the PS. According to the label, the PS contained 250 mg of PAR, 250 mg of acetylsalicylic acid, and 65 mg of CAF. The findings were then compared with results from a validated UV–vis spectrophotometry method, recorded using a GTA—97 UV–vis spectrophotometer (Global, Brazil) (refs (21 and 22)).

## 3 Results and Discussion

### 3.1 Optimizing the Construction and Activation of E-3Ds

#### 3.1.1 E-3D Construction

**Figure 1:**
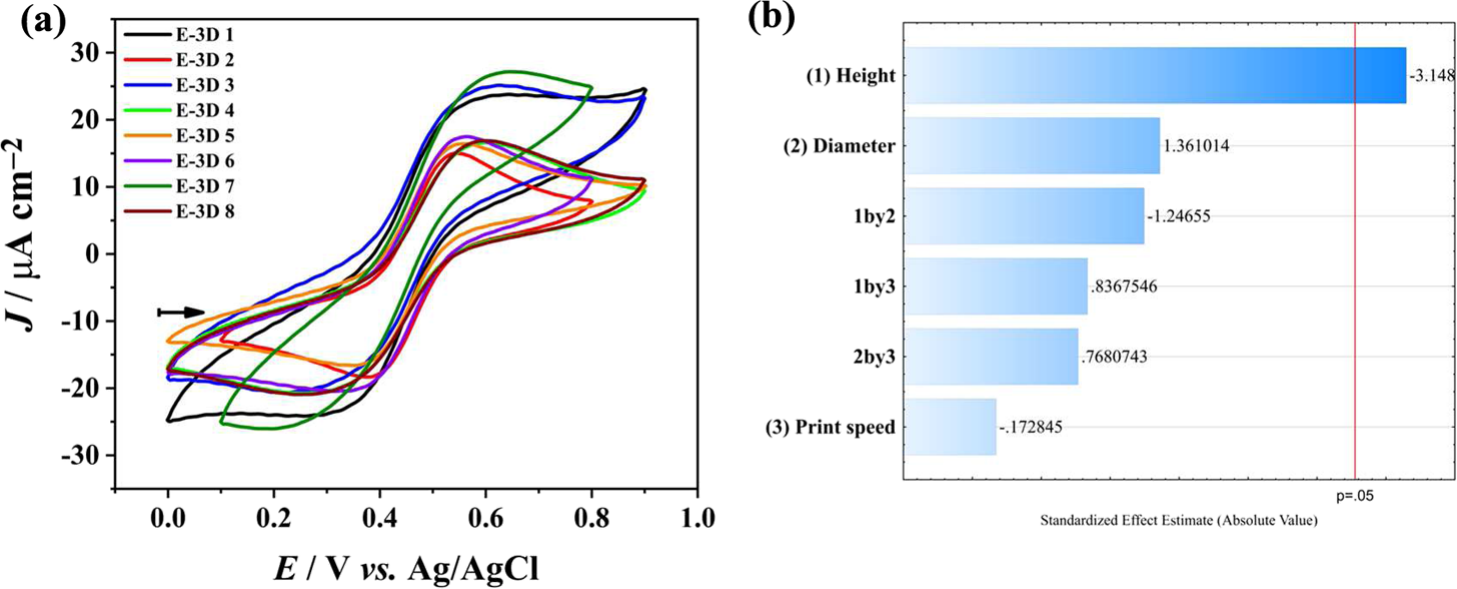
(a) Cyclic voltammograms in the presence of 1.0 mmol L^–1^ of K_3_[Fe(CN)_6_] in 0.5 mol L^–1^ H_2_SO_4_ using different E-3D. Initial potential: 0.0 V; final potential: +0.8 V; scan rate: 50 mV s^–1^. (b) Pareto’s chart showing the significance of the effects calculated for the studied variables: electrode height, electrode diameter, and printing speed.

The design of experiments initially helped in identifying the most significant factors among the variables involved in the 3D printing process. Table S1 in the Supporting Information depicts the current density obtained in each experiment. Analysis of these values determined the effects of electrode height, electrode diameter, and printing speed, as illustrated in Figure 1a. The Pareto chart in Figure 1b highlights the significance of the calculated effects.

Figure 1b demonstrates that only variable 1 (height) was statistically significant. The effect of height was negative, indicating that lower heights resulted in higher current density values. This correlation probably arises because a long electrode increases the electron travel path, leading to higher electrical resistance. In contrast, diameter and printing speed were deemed insignificant within the experimental domain, although they exhibited positive and negative effects, respectively. The effects of all the interactions between the parameters were found to be statistically insignificant within the investigated experimental domain. Therefore, only the height has a significant impact on the electrode construction. Consequently, the optimal experimental conditions for constructing the E-3D sensor were a height of 20 mm, a diameter of 6 mm, and a printing speed of 800 mm min^–1^.

After optimizing the construction, the 3D electrochemical sensor showed a low cost of approximately USD 0.04 (for each electrode).

#### 3.1.2 E-3D Treatment and Activation

**Figure 2:**
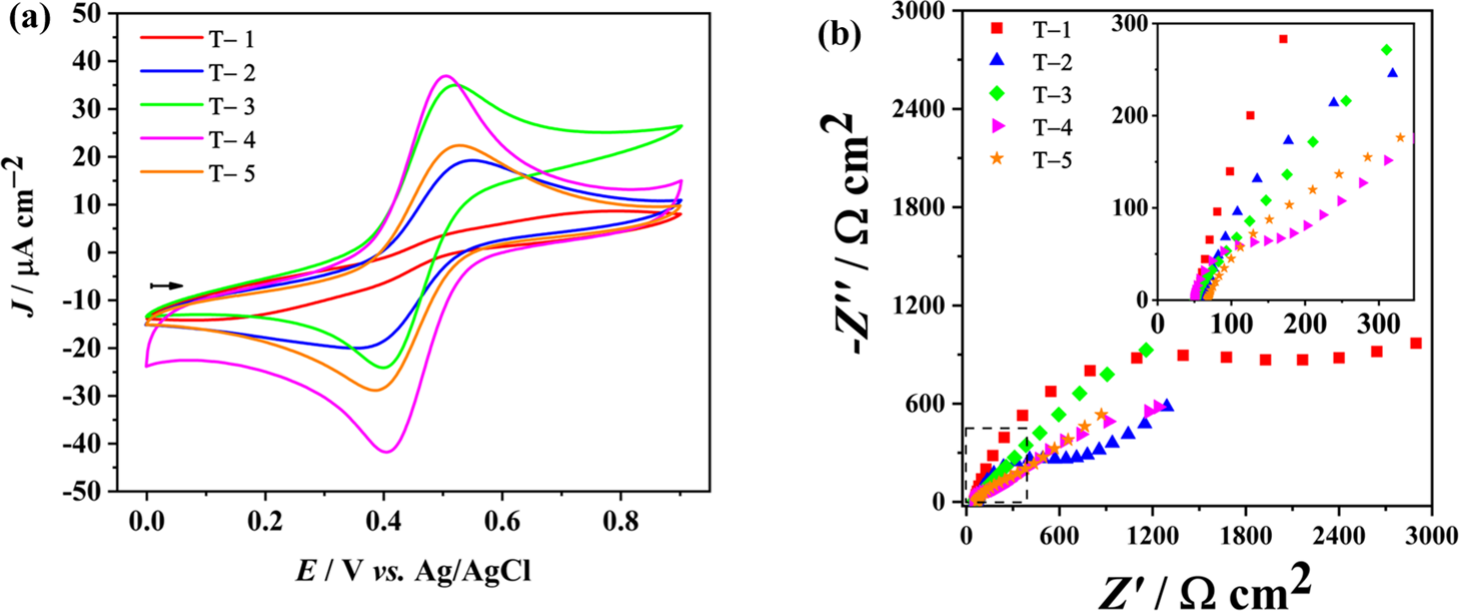
(a) Cyclic voltammograms in the presence of 1.0 mmol L^–1^ of K_3_[Fe(CN)_6_] in 0.5 mol L^–1^ H_2_SO_4_ using the optimized E-3D sensor in different activation forms physical polishing (T-1), physical polishing followed by electrochemical treatment (T-2), physical polishing followed by chemical treatment (T-3), physical polishing followed by chemical and electrochemical treatment (T-4), and physical polishing followed by electrochemical and chemical treatments (T-5). Initial potential: 0.0 V; final potential: +0.9 V; scan rate: 50 mV s^–1^. (b) Impedance diagrams in the complex plane in the presence of 1.0 mmol L^–1^ of K_3_[Fe(CN)_6_] in 0.5 mol L^–1^ H_2_SO_4_ using the optimized E-3D sensor in different activation forms.

After optimizing the construction of the E-3D sensor, five different procedures were studied for treating and activating the surface of the optimized electrode. These treatments comprised physical polishing (T-1), physical polishing followed by electrochemical treatment (T-2), physical polishing followed by chemical treatment (T-3), physical polishing followed by chemical and electrochemical treatment (T-4), and physical polishing followed by electrochemical and chemical treatments (T-5). Figure 2a shows the cyclic voltammograms obtained with different forms of surface treatment and activation on the E-3D. Figure 2b illustrates the impedance diagram in the complex plane for the samples in the different forms of treatment and activation of the E-3D.

As shown in Figure 2a, the T-4 treatment led to higher *I*_peak_ values and Δ*E* values closer to a reversible process (59 mV) following the Nernst equation for the transfer of one electron, as shown in Table 1 (ref (23)). This result is corroborated by the impedance diagrams in the complex plane, which for the same treatment sequence mentioned above, yielded a lower value of charge transfer resistance (*R*_tc_), indicating better electronic transfer between the surface of the E-3D sensor and the solution (vide Table 1).

**Table 1:** Parameters Obtained in the Analysis of the CV and EIS Experiments, Conducted Using 1.0 mmol L^–1^ of K_3_[Fe(CN)_6_] in 0.5 mol L^–1^ H_2_SO_4_

treatment	CV parameters		EIS parameters	
	*I*_ap_ (μA)	*I*_cp_ (μA)	Δ*E* (mV)	*R*_ct_ (kΩ cm^2^)
T-1	2.8	–3.1	435	2.41
T-2	12.5	–11.1	161	0.75
T-3	18.8	–20.7	104	0.22
T-4	24.3	–24.1	92	0.17
T-5	15.8	–1.4	126	0.50

It is important to note that the optimized E-3D treatment and activation condition proposed in this work (T-4) was applied for the simultaneous electroanalysis of paracetamol and caffeine. In the literature, there are various types of treatment and activation of 3D-printed sensors for numerous types of analytes, as shown in Table 2. Cardoso (2020) et al., developed a method of applying +1.4 and 1.0 V sequentially for 200 s for each step using 0.5 mol L^–1^ NaOH solution (ref (2)). However, using the same T-2 treatment a lower CV signal was observed in the presence of 1.0 mmol L^–1^ of K_3_[Fe(CN)_6_] as shown in Figure 2a. Additionally, when coupled with the chemical treatment, a significant improvement was obtained. When associated with chemical treatment (T-4), there was a significant improvement with an increase in the electrochemical signal in the CV and a decrease in the *R*_ct_ as shown in Figure 2b.

**Table 2:** Comparison of Results Obtained in This Work and Reported in the Literature Corresponding to Different Ways of Treating and Activating 3D-Printed Electrochemical Sensors for Different Analytes^a^

electrodes	treatment	procedure	analyte	references
Gpt-PLA	electrochemical	+1.4 V (200 s); –1.0 V (200 s) in 0.5 mol L^–1^ NaOH	UA, DOP, and COV-19	(4)
rGO-PLA	chemical	DMF (15 min), HNO_3_ (15 min), and NaBH_4_ (15 min)	SER and CAT	(24)
Fe(II)-MOF/CB-PLA	electrochemical	–1.4 V (120 s) followed by a DP scan	GLC	(25)
CB-PLA	electrochemical	+1.4 V (200 s); –1.0 V (200 s) in 0.5 mol L^–1^ NaOH	HCQ	(16)
3D-CB/PLA	physical	air plasma jet pen: time (2 min), plasma power (3000 mW)	CPS	(26)
3D-CB/PLA	irradiation and electrochemical	blue-laser power (280 mW); +1.4 V (200 s); –1.0 V (200 s) in 0.5 mol L^–1^ NaOH	HCQ and PRT	(27)
Gph-PLA	chemical and electrochemical	DMF (10 min); –2.5 V (150 s) in PBS (pH 7.2)	[Fe(CN)_6_]^4–/3–^	(28)
CB-PLA	electrochemical	+1.4 V (200 s); –1.0 V (200 s) in 0.5 mol L^–1^ NaOH	Cd^2+^ and Pb^2+^	(29)
CB-PLA	electrochemical	+1.4 V (200 s); –1.0 V (200 s) in 0.5 mol L^–1^ NaOH	TNT	(2)
Gph-PLA	electrochemical	+1.8 V (900 s) in PBS; 0.0 to –1.8 V vs SCE at 50 mV s^–1^	DOP	(30)
CB-PLA			[Ru(NH_3_)_6_]^2+/3+^	(31)
Gpt-PLA	electrochemical	+1.4 V (200 s); –1.0 V (200 s) in 0.5 mol L^–1^ NaOH	CPX	(18)
**CB-PLA**	**physical, chemical, and electrochemical**	**polishing; ultrasonic bath(30 minin0.5 mol L**^**–1**^**NaOH);+1.4 V(200 s);–1.0 V(200 s)in0.5 mol L**^**–1**^**NaOH**	**PAR and CAF**	**this work**

a **Gpt-PLA:** graphite and PLA; **rGO-PLA:** reduced graphene oxide and PLA; **CB-PLA:** carbon black and PLA; **Gph-PLA:** graphene and PLA; **UA:** uric acid; **DOP:** dopamine; **COV-19:** biomarkers of COVID-19; **SER:** serotonin; **CAT:** catechol; **TNT:** 2,4,6-trinitrotoluene; **HCQ:** hydroxychloroquine; **CPS**: capsaicin; **PRT**: paracetamol: **GLC:** glucose; **Cd:** cadmium; **Pb:** lead; **CPX:** ciprofloxacin; **[Fe(CN)**_**6**_**]**^**3–/4–**^**:** ferri/ferrocyanide redox pair; **[Ru(NH**_**3**_**)**_**6**_**]**^**2+/3+**^**:** hexaammineruthenium redox pair; **PAR:** paracetamol; **CAF:** caffeine.

### 3.2 Morphological, Raman Spectroscopy, and Topography Characterization of E-3D

#### 3.2.1 Characterization by SEM

**Figure 3:**
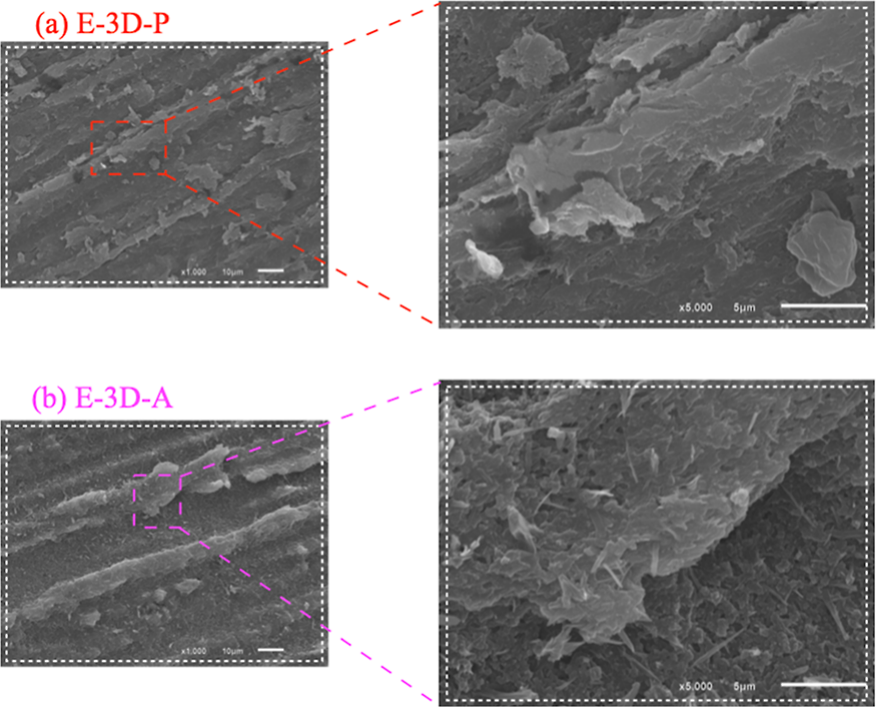
SEM images of electrodes (a) E-3D-P, and (b) E-3D-A.

SEM images reveal grooves on the surfaces of both electrodes, E-3D-P and E-3D-A, as illustrated in Figure 3a,b, respectively. The physically activated electrode, which underwent polishing by sanding, exhibits a surface characterized by grains of irregular sizes and shapes. In contrast, the electrochemically activated electrode displays a more uniform morphology with more regular grains and shapes, resulting in shallower grooves, as shown in Figure 3b. This observation is in line with the AFM images, presented in item 3.2.3, and the electrochemical analysis, which collectively suggest that the electrochemically activated electrode presents a greater exposure of carbon particles on its surface (refs (2, 32–35)).

#### 3.2.2 Characterization by Raman Spectroscopy

The Raman spectra of samples E-3D-P, E-3D-S, and E-3D-A, as depicted in Figure S2 (Supporting Information), display the D and G vibrational modes listed in Table S3 (Supporting Information), which are typical of carbon materials containing graphene-like layers or fragments (such as carbon blacks) (ref (36)).

These observations suggest the presence of defects, such as vacancies, edge sites, and heteroatoms. The low crystallinity of the studied materials indicates the broadening of the D and G bands and the absence of the 2D vibrational mode, consistent with the low crystallinity of carbon black. Another observation is the value of the *I*_D_/*I*_G_ ratio, which did not vary significantly among the studied samples: the found values were 1.0 for E-3D-P, 0.99 for E-3D-S, and 0.95 for E-3D-A. The results indicate that the electrochemical features of E-3D-A are promising due to the procedures used to treat and activate the electrode surface. These procedures may lead to greater carbon exposure, improving its overall performance (refs (7, 34, 37 and 38)).

#### 3.2.3 Topography Characterization and Roughness Parameters by AFM

**Figure 4:**
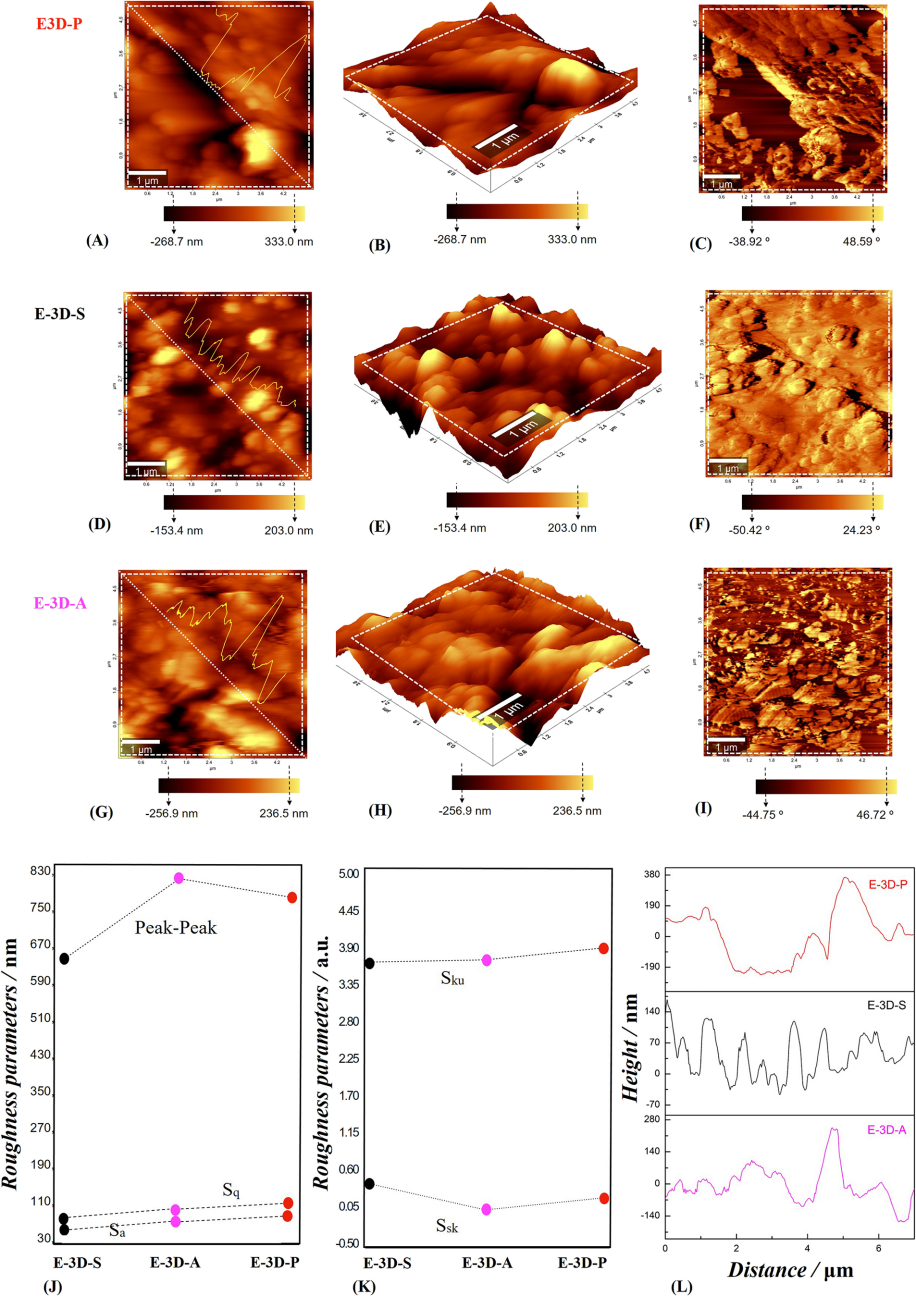
AFM images of the E-3D-P, E-3D-S, and E-3D-A electrodes: 2D and 3D topographic images (a,b,d,e,g,h); phase (c,f,i); roughness parameters *S*_a_, *S*_q_, peak-peak, *S*_sk_ and *S*_ku_ (j,k); diagonal cut curve profile of the electrode topographic images (l).

Images of the surface topography of the E-3D-P, E-3D-S, and E-3D-A electrode systems were recorded using the AFM technique, as shown in Figure 4, where 2D (Figure 4a,c,d,f) and 3D (Figure 4b,e) images of these electrodes are exhibited. Using these topographic images, the electrode roughness parameters were calculated, known as arithmetic mean height (*S*_a_), quadratic mean height (*S*_q_), peak-to-peak height, the surface slope (*S*_sk_), and surface kurtosis (*S*_ku_), respectively, there are shown in Figure 4j,k. An increase in roughness was observed between the E-3D-S and E-3D-A electrodes (*S*_a_: from 56.8 to 73.7 nm; *S*_q_: from 72.9 to 96.3 nm; peak-peak: from 639.8 to 813.6 nm) (refs (35 and 39)).

In contrast, the E-3D-P electrode exhibited higher *S*_a_ (84.5 nm) and *S*_q_ (110.7 nm) values than the E-3D-A electrode. This discrepancy likely results from applying more force than desired during polishing with sandpaper, which removes more CB layers and exposes more of the PLA, as observed via SEM. Two additional parameters, *S*_sk_ and *S*_ku_ were extracted from the topographic images. The values obtained for the electrodes were: for E-3D-S, *S*_sk_ = 0.33 and *S*_ku_ = 3.73; for E-3D-A, *S*_sk_ = −0.08 and *S*_ku_ = 3.76; and for E-3D-P, *S*_sk_ = 0.10 and *S*_ku_ = 3.94, as shown in Figure 4k (refs (35 and 39)).

It was possible to determine the profile of the curve from a diagonal cross-section of each image, as shown in Figure 4l (refs (40–43)). AFM phase imaging, which is sensitive to variations in sample composition, adhesion, friction, and viscoelasticity, proved suitable for distinguishing different components of composite materials. The AFM phase images (Figure 4c,f,i), captured simultaneously with the topographic images (Figure 4a,d,g) of the E-3D-P, E-3D-S, and E-3D-A electrodes, respectively, reveal the porous structure of the PLA film at the interfaces between CB particles or aggregates. In the E-3D-P electrode, which was mechanically polished with sandpaper (Figure 4c), surface nonuniformity is evident, with the PLA film partially covering the CB particles. For the E-3D-S electrode, which underwent no surface treatment, Figure 4f shows that the electrode surface consists mostly of PLA film, with minimal exposure of CB particles. The electrochemically treated E-3D-A electrode (Figure 4i) exhibited uniform exposure of CB particles across the PLA surface, resulting in improved electrochemical performance compared to the other two electrodes, owing to the relatively high electrical conductivity of the CB particles (refs (44–47)).

### 3.3 Application of E-3D Sensor in Electroanalysis of PAR and CAF

#### 3.3.1 Electrochemical Behavior for PAR and CAF

**Figure 5:**
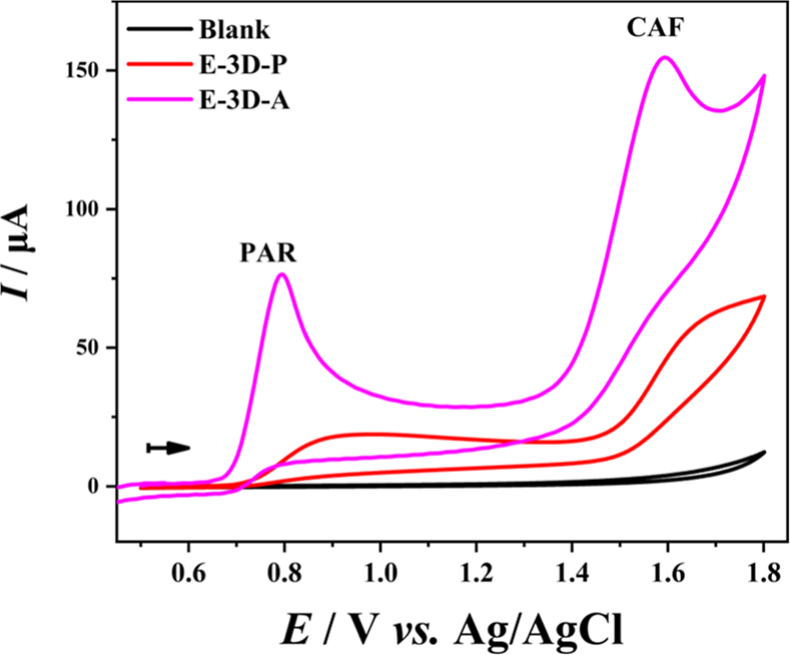
Cyclic voltammograms in the absence (black) and presence of 1.0 mmol L^–1^ of PAR and CAF in 0.5 mol L^–1^ H_2_SO_4_ using E-3D-A (pink), and E-3D-P (red). Initial potential: 0.50 V; final potential: +1.80 V; scan rate: 50 mV s^–1^.

In the study of the activation treatment of the E-3D surface, the process T-4 led to the best responses to [Fe(CN)_6_]^3–^, known as a probe molecule for electrochemical tests. Thus, the analysis of PAR and CAF was carried out to evaluate the effectiveness of the activated 3D-printed electrode (E-3D-A). To this end, the response of the analytes at a concentration of 1.0 mmol L^–1^ in 0.5 mol L^–1^ H_2_SO_4_ was initially studied using CV, as shown in Figure 5. In this test, E-3D-A was used and compared with an electrode that was only physically polished (E-3D-P).

Figure 5 highlights the performance differences between the E-3D-P and E-3D-A electrodes. The CV curve obtained with the E-3D-P electrode revealed two relatively broad peaks corresponding to the irreversible oxidation of PAR (+0.95 V vs. Ag/AgCl) and CAF (+1.70 V vs Ag/AgCl). Conversely, the CV curve recorded with the E-3D-A electrode exhibited two more intense and well-defined peaks at +0.77 V (*vs.* Ag/AgCl) for PAR and +1.60 V (vs Ag/AgCl) for CAF. This improvement reflects the effective activation of the 3D electrode, which enhanced charge transfer and significantly increased the anodic peak current signals for both analytes (refs (2 and 13)).

Carbon materials are well-recognized for their usefulness in CAF electroanalysis (refs (48–50)). These materials are highly valued as electrode matrices due to their robustness, stability in diverse solvents, and broad potential range. For example, graphene and other graphitic materials have demonstrated exceptional sensing capabilities, making them the focus of extensive research. Previous studies showed that reduced graphene oxide (rGO) lowers the oxidation potential for CAF to approximately +1.45 V (vs Ag/AgCl), underscoring rGO’s superior electrocatalytic properties (ref (51)).

Similarly, carbon-based matrices have been extensively used for the development of electrochemical sensors for PAR detection (refs (50, 52–55)). Boumya et al. provided a comprehensive review of carbon-based electrodes for PAR sensing, emphasizing the role of carbon in the oxidation process. However, their work does not explore the E-3D sensors. The E-3D electrode, composed of carbon black, offers a straightforward and cost-effective solution, costing only about USD 0.04 per sensor. Its ease of use and low cost position it as a promising alternative for effective PAR and CAF monitoring in environmental applications.

The electrochemical oxidation mechanisms for PAR and CAF proposed in the literature are illustrated in Scheme S1 (Supporting Information). For PAR, the process involves a two-proton, two-electron exchange leading to the formation of *N*-acetyl-*p*-benzoquinone-imine (refs (54, 56–60)). In the case of CAF, the oxidation occurs in two steps: an initial slow step involving the oxidation of the C-8–N-9 bond via 2H^+^ and 2e^–^, yielding a substituted uric acid, followed by a rapid step that involves the oxidation of this intermediate into a 4,5-diol analog of uric acid through an additional exchange of 2H^+^ and 2e^–^ (refs (51, 60–63)).

##### 3.3.1.1 pH Study

Experiments conducted at different pH values further revealed that in the presence of 0.5 mol L^–1^ H_2_SO_4_ electrolyte, the simultaneous PAR and CAF system produced a higher peak current signal (Figure S3, Supporting Information). For PAR, increasing pH resulted in a decrease in peak current and a shift toward less negative potentials. This behavior did not occur with the electrochemical response of CAF, likely due to its higher p*K*_a_ (approximately 10). Numerous studies in the literature support using H_2_SO_4_ as a supporting electrolyte for drug analysis, corroborating the results shown in Figure S3 (Supporting Information) (refs (22, 51–53, 64–68)).

##### 3.3.1.2 Scan Rate Study

The electrochemical study of mass transport control of the simultaneous oxidation reaction of PAR and CAF using the E-3D-A electrode was evaluated using CV. For this, both analytes in a 0.5 mol L^–1^ H_2_SO_4_ solution were analyzed in different scan rates (*v*), as shown in Figure S4 (Supporting Information). The linearity for anodic peak currents (*I*_p_) vs *v*^1/2^ of PAR and CAF oxidation was *r* = 0.999 (Figure S5, Supporting Information). However, the correlation coefficient between *I*_p_ vs *v* was *r* = 0.981 (Figure S6, Supporting Information). Those values indicate a diffusion-controlled electrochemical behavior on the surface of the 3D sensor. Finally, the plot of log *I*_p_ vs log *v* exhibited a linear relationship (Figure S7, Supporting Information). In both cases, the slope of the curve was lower than 0.50, indicating that the process is predominantly controlled by diffusion (refs (69–72)).

Furthermore, the Laviron equation (eq 1) was applied to determine the number of protons and electrons involved in the oxidation processes for PAR and CAF, offering thoughtful insights into the electrochemical behavior of these analytes (ref (73))
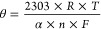
1

Equation 1 is composed by the slope (θ) of the plot of *E*_p_ (peak potential) vs log *v*, obtained from Figure S8; *R* is the universal gas constant (8.314 J mol^–1^ K^–1^); *T* is the temperature (298.5 K); α is the electron transfer coefficient (0.5 for organic compounds) (ref (74)); *n* is the number of transferred electrons; and *F* is the Faraday constant (96,485 C mol^–1^). Using these values in eq 1 revealed that the *n* value was 2.18 and 1.70 for PAR and CAF respectively, indicating that both processes involve 2H^+^ and 2e^–^. For PAR, this value is consistent with the proposed mechanism shown in Scheme S1a. However, in the case of CAF (Scheme S1b), the overall process involved 4H^+^ and 4e^–^, indicating that the reaction rate is primarily limited by the first slow step (ref (75)).

#### 3.3.2 Optimization of DPASV Technique Parameters

Multivariate analysis is a widely applied chemometric tool in various areas of chemistry. One such tool is experimental design, which offers positive aspects over the univariate method, including decreasing the number of experiments and allowing the study of interaction effects. Among these methods, fractional factorial design enables more efficient use of resources by reducing the sample size. This approach is useful when dealing with multiple variables, as it allows for identifying key factors with fewer experiments. However, the drawback is that it provides less detailed information about all possible interactions, making it a balance between efficiency and depth of analysis (refs (76–78)).

In this sense, to select the significant variables for DPASV, the 2^5–1^ fractional design was used. The results obtained in this screening step are shown in Figure S9 (Supporting Information). Observing Figure S9, it is possible to evaluate that the positive effects of *x*_1_ and *x*_2_ (modulation amplitude and step potential, respectively) are the only statistically significant between all the variables and levels.

Therefore, these significant variables were studied in the modeling stage using the FCCD. To perform the FCCD experiments (Table S4, Supporting Information), the chosen unified response was the anodic peak current recorded in the presence of 3.65 and 9.0 μmol L^–1^ of PAR and CAF, respectively, in 0.5 mol L^–1^ H_2_SO_4_. After running all experiments, a second-order polynomial relationship was obtained (eq 2).
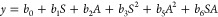
2

For the eq 2, *y* denotes the unified response and the *b*_i_ coefficients relate the anodic peak current with the step potential (*S*) and the modulation amplitude (*A*). The coefficients of the eq 2 were obtained using eq 3 below (refs (76–80)).
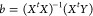
3

**Figure 6:**
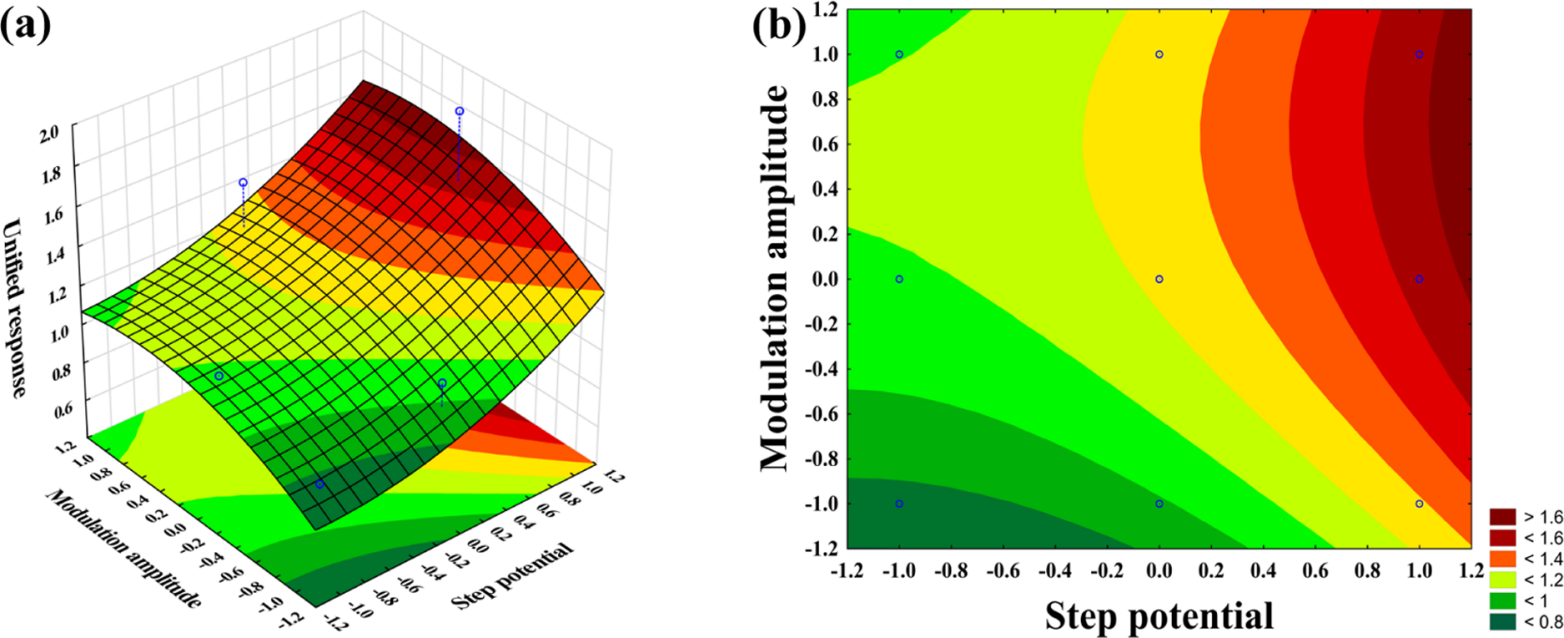
(a) Response surface, and (b) contour plot, showing the dependence of the unified response for step potential and the modulation amplitude.

From the quadratic model obtained in eq 2, it was possible to construct the response surface (Figure 6a), and the level curve (Figure 6b). The significance of the coefficients of the parameters of the quadratic model is shown in the corresponding Pareto graph (Figure S10, Supporting Information). It can be observed that all the effects calculated in eq 2 were significant with 95% confidence, except for the linear interaction between the step potential and the modulation amplitude as illustrated in the Pareto graph (Figure S10, Supporting Information).

The response surface analysis indicates that increasing the modulation amplitude and step potential improves the unified response. A distinct red region on the graph highlights optimal conditions for the DPASV-related variables to maximize the unified response. It can be seen that the condition of Exp 6 (Table S4, Supporting Information) gives the highest value for the unified response. The optimum condition was set at 25 mV (+1) and 80 mV (0) for the step potential and the modulation amplitude, respectively. The other variables, preconcentration time, preconcentration potential, and stirring rate, were set at 150 s, 0.6 V, and 1 rpm, respectively.

Finally, to test the repeatability and stability of the E-3D-A sensor using the optimized DPASV experimental conditions, 25 successive analyses were performed using 50.0 and 100.0 μmol L^–1^ of PAR and CAF in 0.5 mol L^–1^ H_2_SO_4_; the results are shown in Figure S11 (Supporting Information). From these data, it was possible to obtain the values of relative standard deviation, RSD = 0.74% and 3.17% for PAR and CAF, respectively. The RSD values obtained were less than 4.0% for the analytical signals, indicating that the E-3D-A sensor has good stability.

#### 3.3.3 Calibration Curve and Analytical Performance

**Figure 7:**
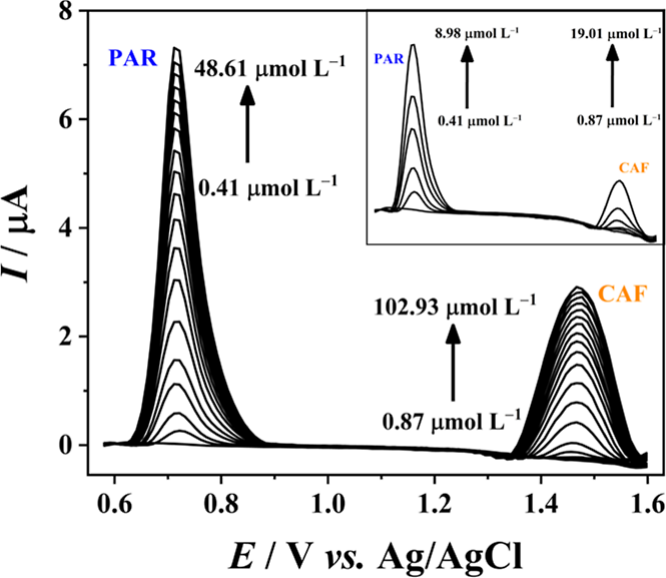
Differential pulse anodic stripping voltammograms with 18 successive additions of PAR and CAF mixed standard solutions in 0.5 mol L^–1^ H_2_SO_4_ using the E-3D-A electrode. DPASV parameters: preconcentration time = 150 s; preconcentration potential = 0.6 V; stirring rate = 1 rpm; step potential = 25 mV; modulation amplitude = 80 mV; start potential = +1.20 V; stop potential = +1.60 V (vs Ag/AgCl). Inset: voltammograms obtained for the first PAR and CAF mixed standard solutions (with the lowest concentrations).

After optimizing the parameters of the DPASV technique, a linearity study was conducted over a wide concentration range of PAR (0.41–48.61 μmol L^–1^) and CAF (0.87–102.93 μmol L^–1^) in 0.5 mol L^–1^ H_2_SO_4_; the obtained differential pulse anodic stripping voltammograms are shown in Figure 7. Calibration curves were then constructed for both drugs, showing two linear ranges with correlation coefficients above 0.99, as illustrated in Figure S12 (Supporting Information). The first linear range (shown in the inset of Figure 7) was defined as the working region for each drug. In this way, it was possible to evaluate the main performance parameters, such as repeatability, limit of detection (LOD), and limit of quantification (LOQ).

To determine the LOD was used the parameters of the calibration curve, applying the equation: LOD = (3 × *s*)/*b*, which *b* is the slope of the calibration curve and *s* is the standard deviation of the intercept. For the LOQ, the equation is LOQ = 3.3 × LOD (refs (76 and 81)). The obtained LOD and LOQ values for PAR were 0.44 and 1.45 μmol L^–1^, respectively, whereas the values LOD = 0.58 μmol L^–1^ and LOQ = 1.92 μmol L^–1^ were found for CAF, in both cases using the E-3D-A sensor.

The method’s accuracy was evaluated in terms of repeatability and intermediate precision, analyzing the performance of the E-3D-A electrode according to the electroanalytical method. The relative standard deviation (RSD %) values obtained for the calibration curve concentrations for both PAR and CAF did not exceed 7.0%, showing that the methodology has good repeatability (refs (76, 82 and 83)).

Subsequently, to investigate the intermediate precision parameter, readings were taken in the presence of 5.0 and 11.0 μmol L^–1^ of PAR and CAF in 0.5 mol L^–1^ H_2_SO_4_ on different days, with an interval of 7 days between tests using the same E-3D-A electrode. Figure S13 (Supporting Information) shows that similar results were obtained in the 2 days, with RSD % of 2.40 and 3.70%, for PAR and CAF, respectively.

Table 3 summarizes the analytical parameters observed using the E-3D-A sensor to simultaneously determine PAR and CAF via DPASV experiments. The performance of the E-3D-A sensor is promising when compared with some of the studies reported in Table 3. It can be seen that similar LOD values were obtained by Lourenção et al., who carried out the simultaneous analysis of PAR and CAF in pharmaceutical formulations and obtained a recovery range from 96.6 to 105.6%; a boron-doped diamond sensor was used in this work, which is a very versatile but extremely expensive sensor (ref (50)).

**Table 3:** Comparison of Analytical Parameters for PAR and CAF Determinations Using the 3D-Printed Sensor Described in This Work with Those Corresponding to Other Similar Sensors Reported in the Literature*

electrode*	method	linear range (μmol L^–1^) PAR/CAF	LOD (μmol L^–1^) PAR/CAF	references
3D-printed sensor	DPV	0–115/0–150	2.84/2.01	(53)
GrRAC	DPV	0–50/0–50	2.01/2.31	(84)
SPCNTE	DPV	0.60–5.30/1.03–56	0.20/0.26	(85)
aGCE	SWV	10–180/10–95	2.55/2.36	(86)
poly(AHNSA)/GCE	SWV	10–125/10–125	0.45/0.79	(60)
BDD	SWV	0.5–830/0.5–830	0.49/0.03	(50)
DLC:VAMWCNT	SWV	1–367/1–917	0.33/0.36	(87)
**E-3D-A**	**DPASV**	**0.4–48/0.9–102**	0.44/0.58	**this work**

* Electrodes: **3D-printed sensor:** 3D-printed sensor constructed using a carbon loaded working electrode; **GrRAC:** sensor fabricated using raw cork (RAC) modified with graphite (Gr). **BDD:** boron-doped diamond-based sensor. **DLC:VAMWCNT:** sensor constructed using vertically aligned multiwalled carbon nanotubes (VAMWCNT) and diamond-like carbon films (DLC).

Katseli et al. developed also an electroanalytical methodology for the simultaneous determination of PAR and CAF in pharmaceutical formulations using 3D-printed sensors (ref (53)). However, the LOD and LOQ values they obtained were higher than those reported here for the E-3D-A sensor. This discrepancy may be attributed to the lack of treatment and activation of the 3D sensor surface.

#### 3.3.4 Quantification of Paracetamol and Caffeine in Real Sample

Finally, the developed 3D-printed sensor was used to determine the PAR and CAF contents in a commercial pharmaceutical sample (PS). After preparing the solution containing PS, an aliquot was taken from the solution and added to the electrochemical cell in 0.5 mol L^–1^ H_2_SO_4_. The voltammogram of the PS sample reading is shown in Figure S14a (Supporting Information). The recovery values obtained for PAR and CAF were 97% and 104%, respectively, which were consistent with the values reported in the PS label. Therefore, the proposed method using the E-3D-A sensor proved to be highly accurate.

The results obtained from the UV–vis method demonstrated higher recoveries of PAR (469%) and CAF (230%) compared to the proposed electroanalytical method using the E-3D-A described in this work (Figure S14b, Supporting Information). This finding indicates that in UV–vis measurements there is interference from other chemical substances, and can impair the accuracy of UV–vis analysis. This finding indicates that the UV–vis measurements might be affected by interference from other chemical substances (ref (21)), which can limit the accuracy of the contents derived using that method, which is not the case of the electroanalytical method reported here.

## 4 Conclusions

This work presents an optimized 3D-printed electrochemical sensor with a manufacturing cost of USD 0.04. The study emphasizes that effective treatment and activation of the electrode surface are critical for improving the electroanalytical performance. The electrode activation methodology, which involves sequential physical polishing, ultrasonic bath with NaOH solution, and electrochemical treatment, significantly enhances the sensor performance. The optimized E-3D-A sensor demonstrates increased sensitivity for the simultaneous analysis of PAR and CAF. The design of experiments played a crucial role in fine-tuning the DPASV parameters, resulting in low values of LOD (0.44 μmol L^–1^ for PAR and 0.58 μmol L^–1^ for CAF) and LOQ (1.45 μmol L^–1^ for PAR and 1.92 μmol L^–1^ for CAF) in a 0.5 mol L^–1^ H_2_SO_4_ medium. The developed methodology effectively quantifies PAR and CAF in pharmaceutical samples, with recovery rates ranging from 97 to 104%. Additionally, the electrode exhibits high analytical stability, with relative standard deviations of 3.17 and 0.74% for PAR and CAF, respectively. Overall, this simple, low-cost methodology offers robust and reliable performance for electrochemical sensing applications, allowing the monitoring, and the quantification of PAR and CAF present in pharmaceutical formulations.

## Supporting Information Available

Additional results are presented in the following figures, tables, and scheme: Figure S1. Image of the electrochemical cell system used in the study. Working electrode (W.E.): E-3D, reference electrode (R.E.): Ag/AgCl (3.0 mol L^–1^ KCl) and auxiliary electrode (A.E.): platinum bar. Figure S2. Raman spectra obtained for the 3D-printed electrodes: (red) E-3D-P (sandpaper polishing treatment), (black) E-3D-S (without treatment), and (pink) E-3D-A (electrochemical treatment). Figure S3. Cyclic voltammograms in the absence and presence of 1.0 mmol L^–1^ of PAR and CAF in 0.5 mol L^–1^ H_2_SO_4_ using (pink) E-3D-A, and (red) E-3D-P. Initial potential: 0.50 V; final potential: +1.80 V; scan rate: 50 mV s^–1^. Figure S4. Cyclic voltammograms at scan rates ranging from 10 to 200 mV s^–1^ on E-3D-A for 1.0 × 10^–3^ mol L^–1^ PAR, and CAF in 0.5 mol L^–1^ H_2_SO_4_. Figure S5. Linear fitting obtained for the *I*_p_ vs *v*^1/2^ data for PAR (blue), and CAF (yellow). Figure S6. Linear fitting obtained for the *I*_p_ vs *v* data for PAR (blue), and CAF (yellow). Figure S7. Linear fitting obtained for the log *I*_p_ vs log *v* data for PAR (blue), and CAF (yellow). Figure S8. Anodic peak potentials versus logarithm of the scan rate for PAR (blue), and CAF (yellow). Figure S9. Pareto’s chart showing the significance of the effects calculated for the variables studied in the 2^5–1^ fractional factorial design. Figure S10. Pareto’s graph showing the significance of the coefficients of the parameters of the quadratic model used in the FCCD of the DPASV variables. Figure S11. (a) Differential pulse anodic stripping voltammograms for the study of stability in the presence of 50.0 μmol L^–1^ PAR and 100.0 μmol L^–1^ CAF in 0.5 mol L^–1^ H_2_SO_4_. Variation of *I*_p_ as a function of the number of measurements (*n* = 25) using E-3D-A for PAR (b) and CAF (c). DPASV parameters: preconcentration time = 150 s; preconcentration potential = −0.60 V; stirring rate = 1 rpm; step potential = 25 mV; modulation amplitude = 80 mV; start potential = +1.20 V; stop potential = +1.60 V (vs Ag/AgCl). Figure S12. Calibration curves for PAR (a) and CAF (b) with 18 successive additions of PAR and CAF mixed standard solutions using E-3D-A in 0.5 mol L^–1^ H_2_SO_4_. Figure S13. Study of intermediate precision parameter in the presence of 5.0 and 11.0 μmol L^–1^ of PAR and CAF in 0.5 mol L^–1^ H_2_SO_4_ on different days, with an interval of 7 days between tests using the same E-3D-A. Figure S14. (a) Differential pulse anodic stripping voltammogram recorded for the pharmaceutical sample (PS). DPASV parameters: preconcentration time = 150 s; preconcentration potential = −0.60 V; stirring rate = 1 rpm; step potential = 25 mV; modulation amplitude = 80 mV; start potential = +1.20 V; stop potential = +1.60 V (*vs*. Ag/AgCl). (b) UV–Vis spectra recorded for the indicated samples. Table S1. Matrix of experiments of the 2^3^ type for the parameters electrode height, electrode diameter, and printing speed. Table S2. Matrix of experiments for the variables studied in the 2^5–1^ fractional factorial design. Unified response is relative to the presence of 3.65 μmol L^–1^ of PAR and 9.0 μmol L^–1^ of CAF. Table S3 Assignments of the bands identified in the Raman spectra shown in Figure S1, corresponding to E-3D-P, E-3D-S and E-3D-A electrodes. Table S4—FCCD design matrix for variables and their real and coded levels used in the optimization of DPV parameters. Unified response is relative to the presence of 3.65 μmol L^–1^ of PAR and 9.0 μmol L^–1^ of CAF. Scheme S1. Proposed mechanisms for electrochemical oxidation in acid medium for PAR (refs (54, 56–60)) (a), and CAF (b) (PDF)
