# Supplementary material for: Optimizing the Construction and Activation of 3D-Printed Electrochemical Sensors: An Experimental Design Approach for Simultaneous Electroanalysis of Paracetamol and Caffeine
Source: ACS Omega. 2025 Jan 6;10(1):1131–43. doi: 10.1021/acsomega.4c08593 (PMC11740634; doi:10.1021/acsomega.4c08593)
Supplement: Supplementary file 1 — ao4c08593_si_001.pdf [file ao4c08593_si_001.pdf]

Optimizing the construction and activation of 3D-printed electrochemical sensors: an experimental design approach for simultaneous electroanalysis of paracetamol and caffeine

José G. A. Rodrigues[a,b*], Tárcila M. N. Silva[b], Sidnei B. G. Junior[b], Antônio A. L. Marins[c], Gabriel F. S. Santos[d], Rafael Q. Ferreira[b], Jair C. C. Freitas[a].

[a]Laboratory of Carbon and Ceramic Materials (LMC), Department of Physics, Center of Exact Sciences, Federal University of Espírito Santo, Vitória, ES, Brazil.

[b]Electrochemistry Research and Development Laboratory, Department of Chemistry, Center of Exact Sciences, Federal University of Espírito Santo, Vitória, ES, Brazil.

[c]Multiuser Instrumentation Laboratory, Center of Exact Sciences, Federal University of Espírito Santo, Vitória, ES, Brazil.

[d]Center of Research, Innovation and Development of Espirito Santo, Ladeira Eliezer Batista, Cariacica, ES, Brazil.

*E-mail: jose.g.rodrigues@ufes.br and zeka6@hotmail.com

Supporting Information

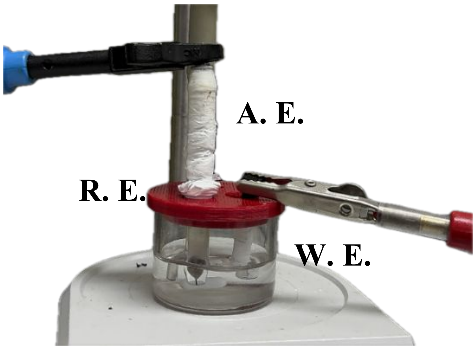

Figure S1. Image of the electrochemical cell system used in the study. Working electrode (W.E.): E-3D, reference electrode (R.E.): Ag/AgCl (3.0 mol L^{-1} KCl) and auxiliary electrode (A.E.): platinum bar.

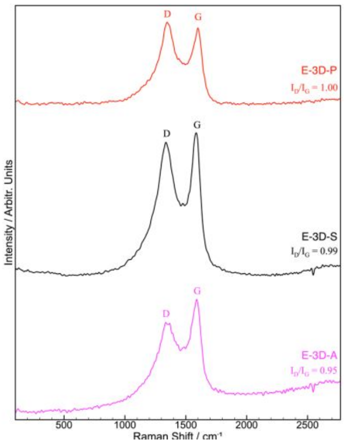

Figure S2. Raman spectra obtained for the 3D-printed electrodes: (—) E-3D-P (sandpaper polishing treatment), (—) E-3D-S (without treatment), and (—) E-3D-A (electrochemical treatment).

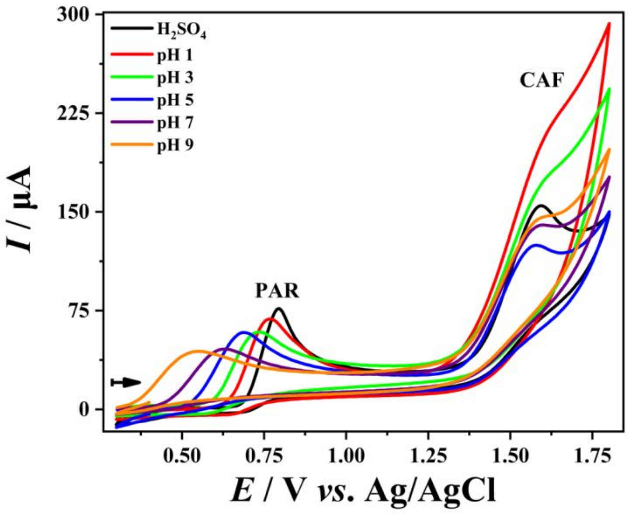

Figure S3. Cyclic voltammograms in the absence and presence of 1.0 mmol L^{-1} of PAR and CAF in 0.5 mol L^{-1} H_2SO_4 using (—) E-3D-A, and (—) E-3D-P. Initial potential: 0.50 V; final potential: + 1.80 V; scan rate: 50 mV s^{-1}.

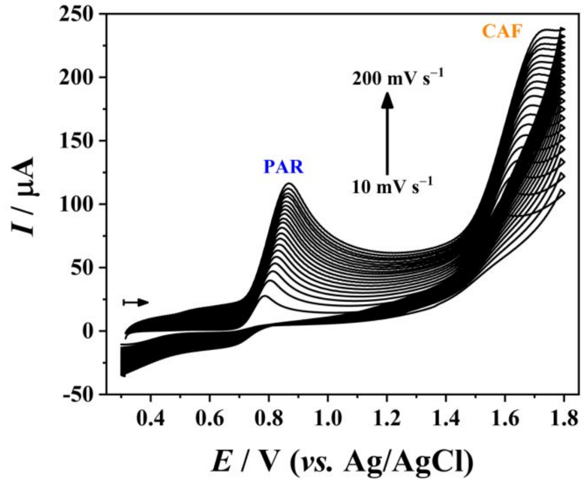

Figure S4. Cyclic voltammograms at scan rates ranging from 10 to 200 mV s^{-1} on E-3D-A for 1.0×10^{-3} mol L^{-1} PAR, and CAF in 0.5 mol L^{-1} H_2SO_4.

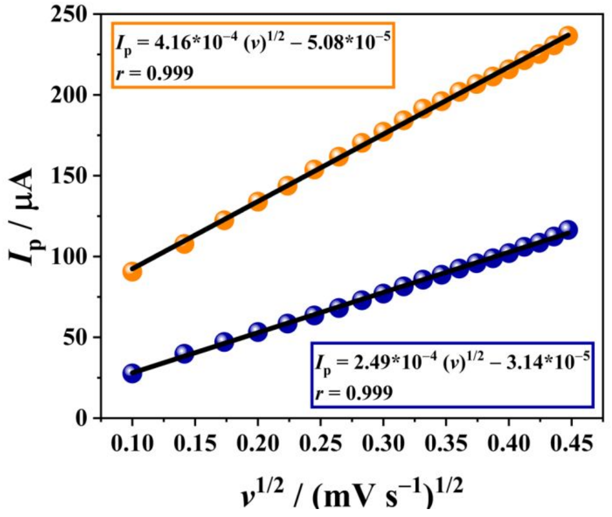

Figure S5. Linear fitting obtained for the I_p *vs.* $v^{1/2}$ data for PAR (—), and CAF (—).

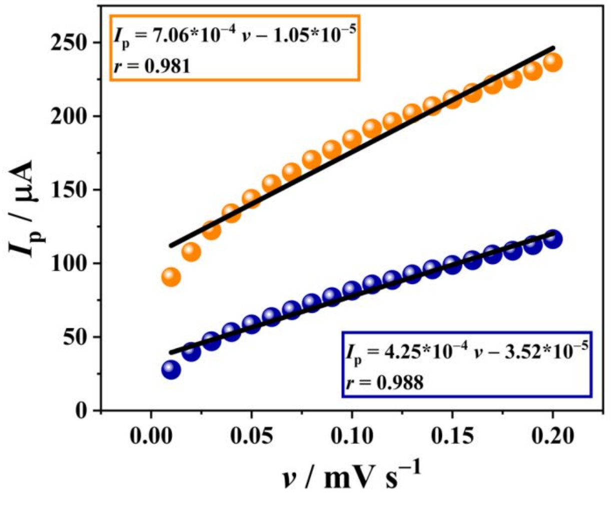

Figure S6. Linear fitting obtained for the I_p *vs.* v data for PAR (—), and CAF (—).

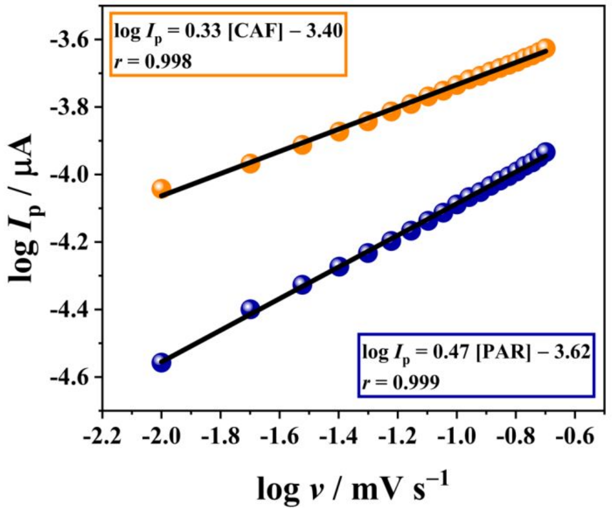

Figure S7. Linear fitting obtained for the log I_p *vs.* log v data for PAR (—), and CAF (—).

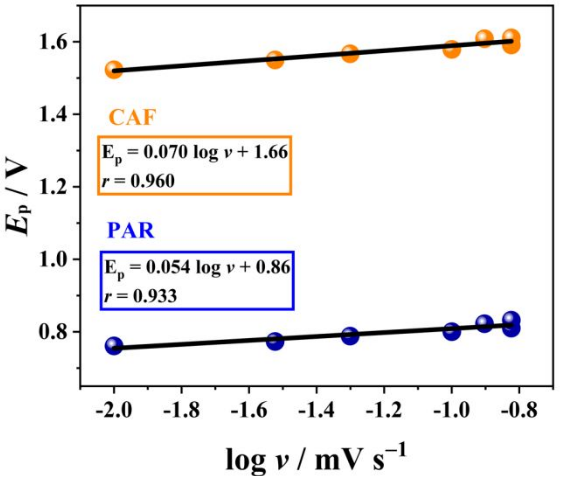

Figure S8. Anodic peak potentials *versus* logarithm of the scan rate for PAR (—), and CAF (—).

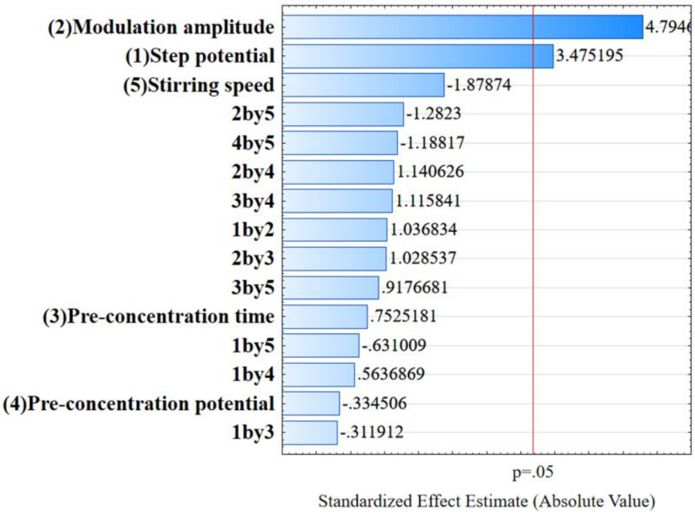

Figure S9. Pareto´s chart showing the significance of the effects calculated for the variables studied in the 2^{5-1} fractional factorial design.

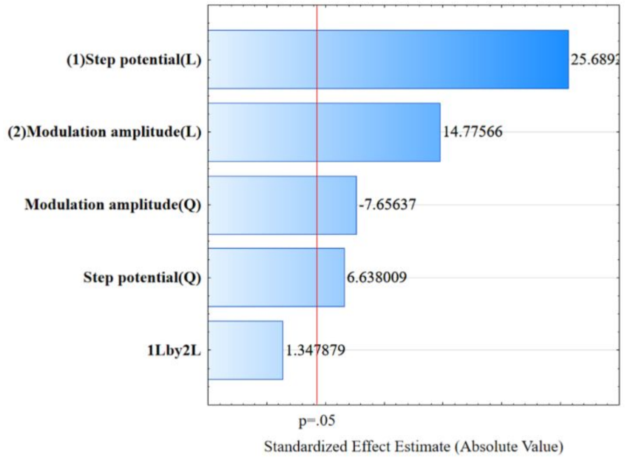

Figure S10. Pareto's graph showing the significance of the coefficients of the parameters of the quadratic model used in the FCCD of the DPASV variables.

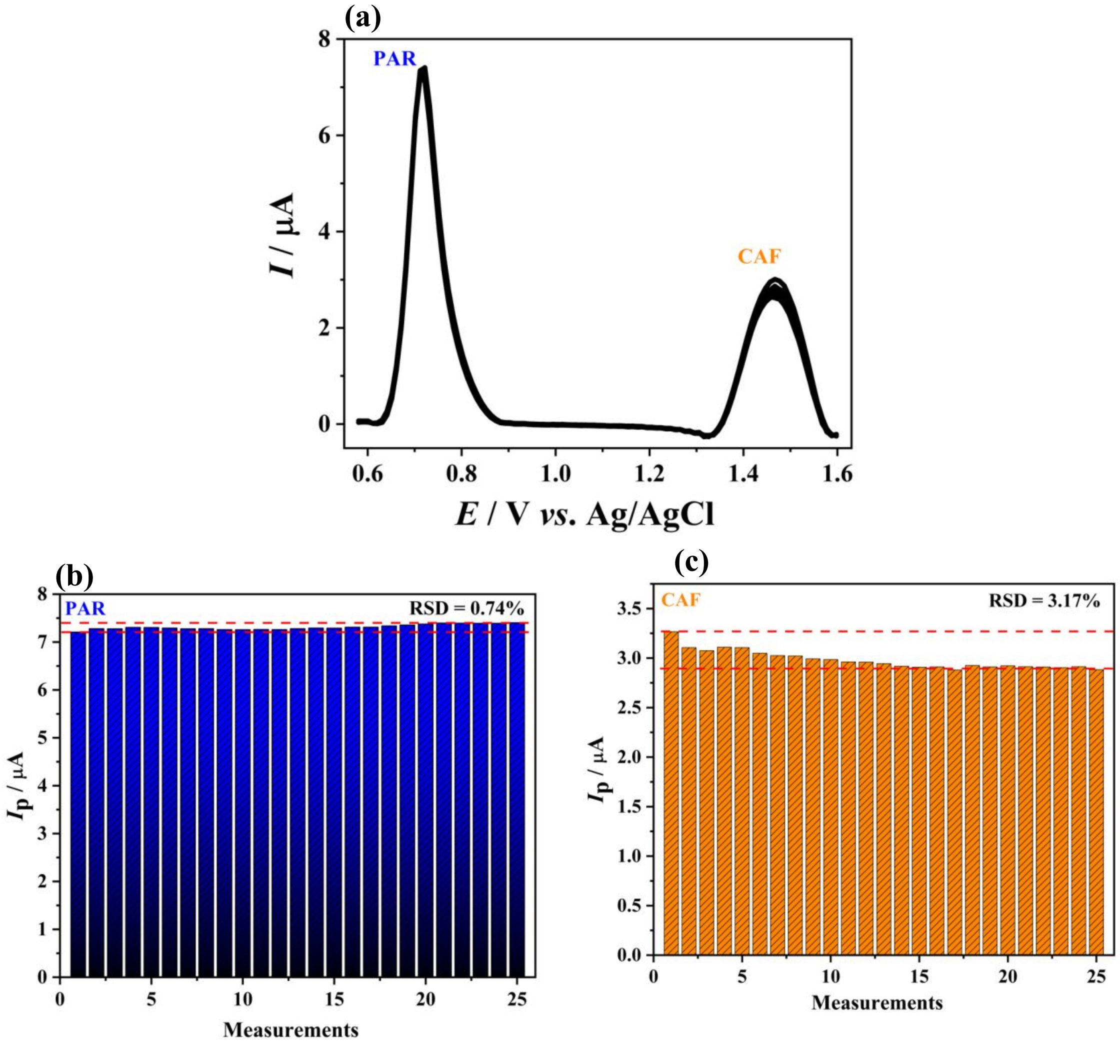

Figure S11. (a) Differential pulse anodic stripping voltammograms for the study of stability in the presence of 50.0 μmol L^{-1} PAR and 100.0 μmol L^{-1} CAF in 0.5 mol L^{-1} H_2SO_4. Variation of I_p as a function of the number of measurements (n = 25) using E-3D-A for PAR **(b)** and CAF **(c)**. DPASV parameters: pre-concentration time =

150 s; pre-concentration potential = −0.60 V; stirring rate = 1 rpm; step potential = 25 mV; modulation amplitude = 80 mV; start potential = +1.20 V; stop potential = +1.60 V (*vs.* Ag/AgCl).

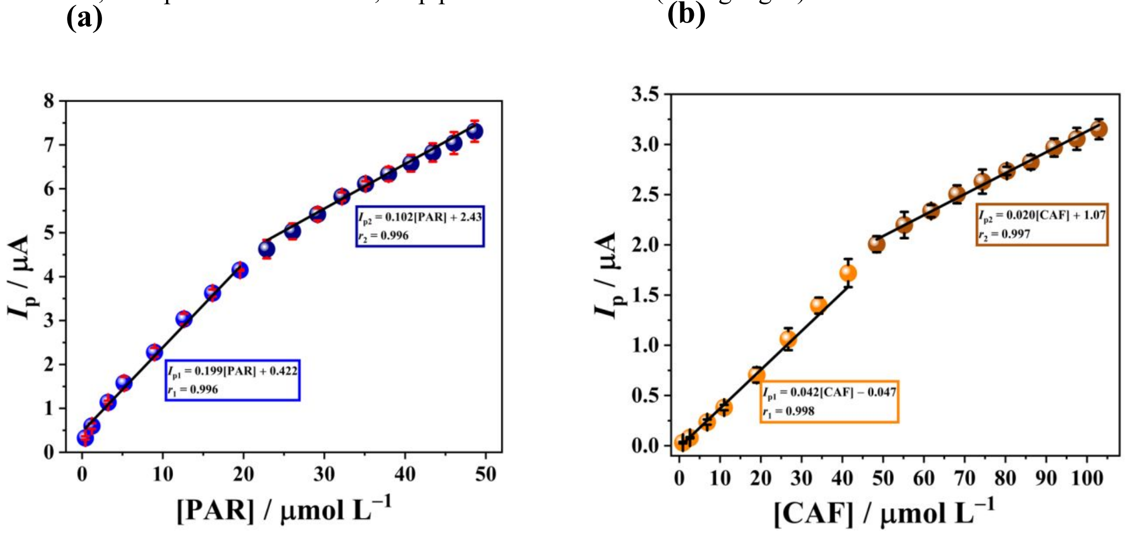

Figure S12. Calibration curves for PAR **(a)** and CAF **(b)** with 18 successive additions of PAR and CAF mixed standard solutions using E-3D-A in 0.5 mol L^{-1} H_2SO_4.

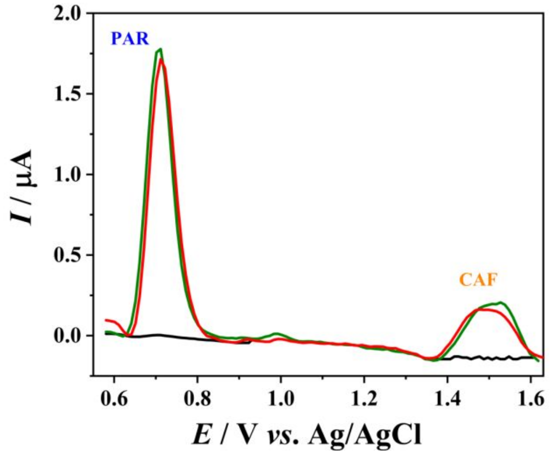

Figure S13. Study of intermediate precision parameter in the presence of 5.0 and 11.0 μmol L^{-1} of PAR and CAF in 0.5 mol L^{-1} H_2SO_4 on different days, with an interval of seven days between tests using the same E-3D-A.

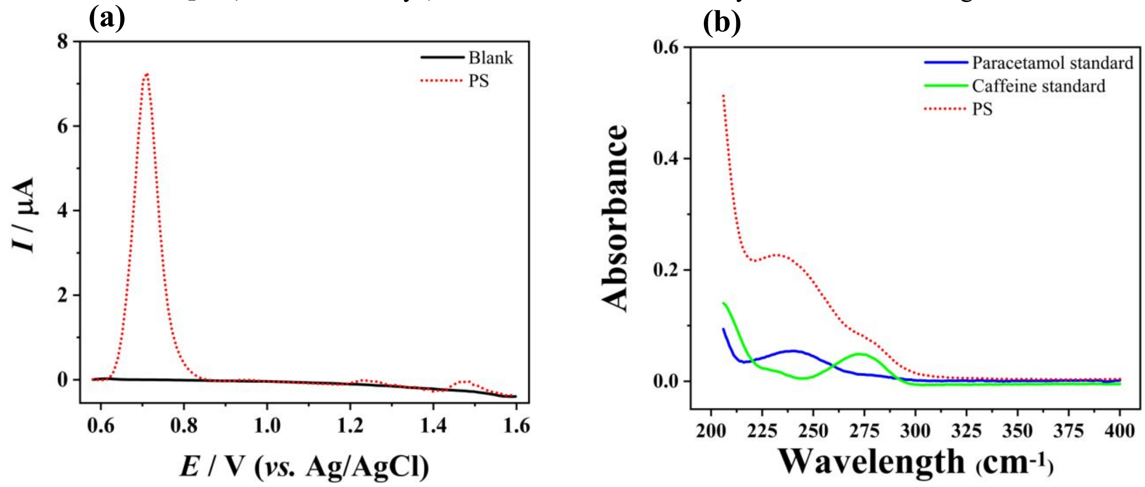

Figure S14. (a) Differential pulse anodic stripping voltammogram recorded for the pharmaceutical sample (PS). DPASV parameters: pre-concentration time = 150 s; pre-concentration potential = −0.60 V; stirring rate = 1 rpm; step potential = 25 mV; modulation amplitude = 80 mV; start potential = +1.20 V; stop potential = +1.60 V (*vs.* Ag/AgCl)). **(b)** UV-Vis spectra recorded for the indicated samples.

Table 1S. Matrix of experiments of the 2^3 type for the parameters electrode height, electrode diameter, and printing speed.

**Exp**	**Height (mm)**	**Diamete r (mm)**	**Printing speed (mm min^{-1})**
**1**	20 (−1)	3 (−1)	400 (−1)
**2**	20 (−1)	3 (−1)	800 (1)
**3**	20 (−1)	6 (1)	400 (−1)
**4**	20 (−1)	6 (1)	800 (1)
**5**	40 (1)	3 (−1)	400 (−1)
**6**	40 (1)	3 (−1)	800 (1)
**7**	40 (1)	6 (1)	400 (−1)
**8**	40 (1)	6 (1)	800 (1)
**9**	30 (0)	4.5 (0)	600 (0)
**10**	30 (0)	4.5 (0)	600 (0)
**11**	30 (0)	4.5 (0)	600 (0)

Table S2. Matrix of experiments for the variables studied in the 2^{5-1} fractional factorial design. Unified response is relative to the presence of 3.65 µmol L^{-1} of PAR and 9.0 µmol L^{-1} of CAF.

**Exp**	**Step potential (mV) (x_1)**	**Modulation amplitude (mV) (x_2)**	**Pre-conc. time (s) (x_3)**	**Pre-conc. potential (V) (x_4)**	**Stirring rate (rpm) (x_5)**	**Unified response (y)**
**1**	5 (−1)	30 (−1)	90 (−1)	0.6 (−1)	3 (+1)	0.66
**2**	15 (+1)	30 (−1)	90 (−1)	0.6 (−1)	1 (−1)	0.95
**3**	5 (−1)	70 (+1)	90 (−1)	0.6 (−1)	1 (−1)	0.95
**4**	15 (+1)	70 (+1)	90 (−1)	0.6 (−1)	3 (+1)	1.11
**5**	5 (−1)	30 (−1)	150 (+1)	0.6 (−1)	1 (−1)	0.40
**6**	15 (+1)	30 (−1)	150 (+1)	0.6 (−1)	3 (+1)	0.75
**7**	5 (−1)	70 (+1)	150 (+1)	0.6 (−1)	3 (+1)	0.91
**8**	15 (+1)	70 (+1)	150 (+1)	0.6 (−1)	1 (−1)	1.44
**9**	5 (−1)	30 (−1)	90 (−1)	0.4 (+1)	1 (−1)	0.41
**10**	15 (+1)	30 (−1)	90 (−1)	0.4 (+1)	3 (+1)	0.48
**11**	5 (−1)	70 (+1)	90 (−1)	0.4 (+1)	3 (+1)	0.46
**12**	15 (+1)	70 (+1)	90 (−1)	0.4 (+1)	1 (−1)	1.67
**13**	5 (−1)	30 (−1)	150 (+1)	0.4 (+1)	3 (+1)	0.41
**14**	15 (+1)	30 (−1)	150 (+1)	0.4 (+1)	1 (−1)	0.81
**15**	5 (−1)	70 (+1)	150 (+1)	0.4 (+1)	1 (−1)	1.25
**16**	15 (+1)	70 (+1)	150 (+1)	0.4 (+1)	3 (+1)	1.40
**17**	10 (0)	50 (0)	120 (0)	0.5 (0)	2 (0)	0.61
**18**	10 (0)	50 (0)	120 (0)	0.5 (0)	2 (0)	0.62
**19**	10 (0)	50 (0)	120 (0)	0.5 (0)	2 (0)	0.67

Table. S3 Assignments of the bands identified in the Raman spectra shown in Fig. S1, corresponding to E-3D-P, E-3D-S and E-3D-A electrodes.

	**Assignments**				
	**Red Spectrum E-3D-P (cm^{-1})**	**Black Spectrum E-3D-S (cm^{-1})**	**Magenta Spectrum E-3D-A (cm^{-1})**	**Literature data (cm^{-1})**	
D	1347	1335	1335	1349	(Ref 36)
				1356	(Ref 7)
				1320, 1325, 1330, 1335, 1340, 1370	(Ref 39)
				1341, 1355, 1357	(Ref 40)
G	1600	1592	1584	1571	(Ref 36)
				1600	(Ref 7)
				1580, 1585, 1600	(Ref 39)
				1600	(Ref 40)
I_D/I_G	1.00	0.99	0.95		

Table S4 - FCCD design matrix for variables and their real and coded levels used in the optimization of DPV parameters. Unified response is relative to the presence of 3.65 µmol L^{-1} of PAR and 9.0 µmol L^{-1} of CAF.

**Exp**	**Step potential (mV) (x_1)**	**Modulation amplitude (mV) (x_2)**	**Unified response (y)**
**1**	15 (−1)	70 (−1)	0.895
**2**	25 (+1)	70 (−1)	1.141
**3**	15 (−1)	90 (+1)	1.069
**4**	25 (+1)	90 (+1)	1.373
**5**	15 (−1)	80 (0)	1.086
**6**	25 (+1)	80 (0)	1.884
**7**	20 (0)	70 (−1)	1.109
**8**	20 (0)	90 (+1)	1.477
**9**	20 (0)	80 (0)	1.091
**10**	20 (0)	80 (0)	1.129
**11**	20 (0)	80 (0)	1.093

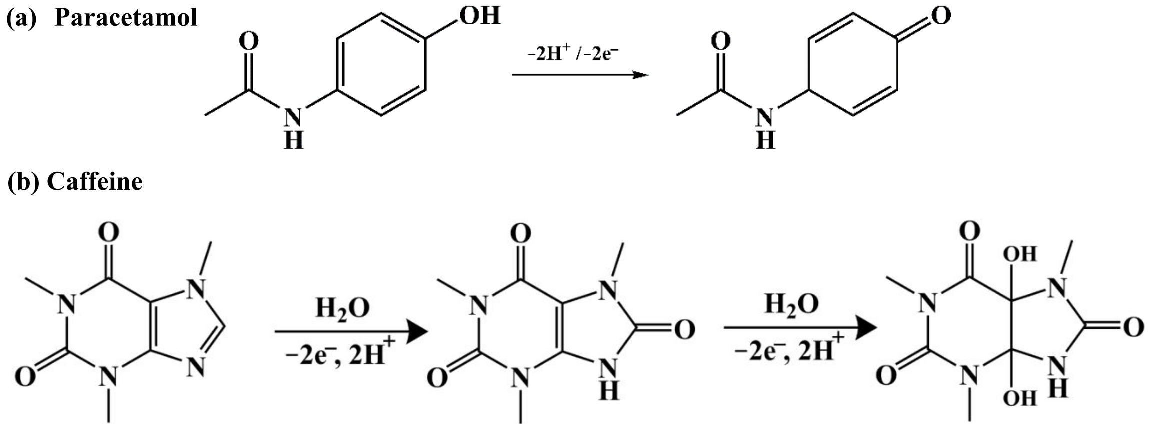

Scheme S1. Proposed mechanisms for electrochemical oxidation in acid medium for PAR (Ref 54,56–60) **(a)**, and CAF **(b)**, (Ref 51,60–63).

References

51. J.G.A. Rodrigues, M.G.S. Barreto, S.B. Gomes Junior, T.M.N. da Silva, A.A.L. Marins, G.F.S. dos Santos, G.L. Carvalho, J.V.B. Del Piero, F.C.A. Silva, T.L.G. Costa, W.L. Scopel, R.Q. Ferreira, and J.C.C. Freitas, Study of the Interaction between Caffeine and Graphenic Materials Employing Computational Analysis and Electrochemistry, *J. Solid State Electrochem.*, Springer Berlin Heidelberg, 2024, doi:10.1007/s10008-024-05958-8.

54. W. Boumya, N. Taoufik, M. Achak, and N. Barka, Chemically Modified Carbon-Based Electrodes for the Determination of Paracetamol in Drugs and Biological Samples, *J. Pharm. Anal.*, 2021, **11**(2), p 138–154.

56. R.N. Goyal and S.P. Singh, Voltammetric Determination of Paracetamol at C60-Modified Glassy Carbon Electrode, *Electrochim. Acta*, 2006, **51**(15), p 3008–3012.

57. Y. Fan, J.H. Liu, H.T. Lu, and Q. Zhang, Electrochemical Behavior and Voltammetric Determination of Paracetamol on Nafion/TiO2-Graphene Modified Glassy Carbon Electrode, *Colloids Surfaces B Biointerfaces*, Elsevier B.V., 2011, **85**(2), p 289–292, doi:10.1016/j.colsurfb.2011.02.041.

58. M.M. Patil, N.P. Shetti, S.J. Malode, D.S. Nayak, and T.R. Chakklabbi, Electroanalysis of Paracetamol at Nanoclay Modified Graphite Electrode, *Mater. Today Proc.*, Elsevier Ltd., 2019, **18**, p 986–993, doi:10.1016/j.matpr.2019.06.538.

59. N.K. Vasantakumarnaik, G. Krishnamurthy, M. Pari, N.S. Kumar, N. Venugopal, and T. Manjuraj, Synthesis, Physicochemical Characterisation of Novel Azo-2-{[(E)-{2-

Hydroxy-3-Methoxy-5-[(E)-(4-Nitrophenyl)Diazenyl]Phenyl}methylidene]Amino}pyridin-3-Ol and Its Metal Complexes; Evaluation of Anti-Diabetic, Anti-Tuberculosis, DFT, Docking and Electroche, *J. Mol. Struct.*, Elsevier B.V., 2025, **1322**(P3), p 140461, doi:10.1016/j.molstruc.2024.140461.

60. M. Tefera, A. Geto, M. Tessema, and S. Admassie, Simultaneous Determination of Caffeine and Paracetamol by Square Wave Voltammetry at Poly(4-Amino-3-Hydroxynaphthalene Sulfonic Acid)-Modified Glassy Carbon Electrode, *Food Chem.*, Elsevier Ltd, 2016, **210**, p 156–162, doi:10.1016/j.foodchem.2016.04.106.

61. J. Wang, F. Yin, W. Tang, N. Zhang, L. Li, S. Zheng, J. Tang, and J. Guo, Electrochemical Detection of Acetaminophen and Caffeine Using Ag Nanoparticles Doped Metal-Organic Framework (ZIF-67) Composites, *Int. J. Electrochem. Sci.*, Elsevier B.V., 2023, **18**(11), p 100334, doi:10.1016/J.IJOES.2023.100334.

62. N.A. Nia, M.M. Foroughi, and S. Jahani, Simultaneous Determination of Theobromine, Theophylline, and Caffeine Using a Modified Electrode with Petal-like MnO2 Nanostructure, *Talanta*, Elsevier B.V., 2021, **222**(May 2020), p 121563, doi:10.1016/j.talanta.2020.121563.

63. M.K.S. Monteiro, S.S.M. Paiva, D.R. da Silva, V.J.P. Vilar, C.A. Martínez-Huitle, and E. V. dos Santos, Novel Cork-Graphite Electrochemical Sensor for Voltammetric Determination of Caffeine, *J. Electroanal. Chem.*, Elsevier, 2019, **839**(March), p 283–289, doi:10.1016/j.jelechem.2019.03.030.
